# 2-(2,3,4,9-Tetra­hydro-1*H*-carbazol-1-ylidene)propane­dinitrile

**DOI:** 10.1107/S1600536810022671

**Published:** 2010-06-18

**Authors:** R. Archana, K. Prabakaran, K. J. Rajendra Prasad, A. Thiruvalluvar, R. J. Butcher

**Affiliations:** a PG Research Department of Physics, Rajah Serfoji Government College (Autonomous), Thanjavur 613 005, Tamilnadu, India; b Department of Chemistry, Bharathiar University, Coimbatore 641 046, Tamilnadu, India; c Department of Chemistry, Howard University, 525 College Street NW, Washington, DC 20059, USA

## Abstract

In the title mol­ecule, C_15_H_11_N_3_, the dihedral angle between the benzene ring and the fused pyrrole ring is 1.07 (5)°. The cyclo­hexene ring adopts an envelope conformation: the dicyano­methyl­ene group at position 1 has a coplanar orientation. An intra­molecular N—H⋯N hydrogen bond generates an *S*(7) ring motif. Inter­molecular N—H⋯N hydrogen bonds form an *R*_2_^2^(14) ring in the crystal. A C—H⋯π inter­action involving the benzene ring is also found in the structure.

## Related literature

For naturally occurring carbazole alkaloids see: Scott *et al.* (2006 ▶). For the biological activity of carbazole alkaloids see: Ramsewak *et al.*(1999 ▶); Tachibana *et al.* (2001 ▶); Nakahara *et al.* (2002 ▶). For the crystal structures of substituted carbazole derivatives see: Gunaseelan *et al.* (2007*a* ▶,*b* ▶, 2009 ▶); Thiruvalluvar *et al.* (2007 ▶); Sridharan *et al.* (2008 ▶). For ring conformations, see: Cremer & Pople (1975 ▶). For hydrogen-bond motifs, see: Bernstein *et al.* (1995 ▶).
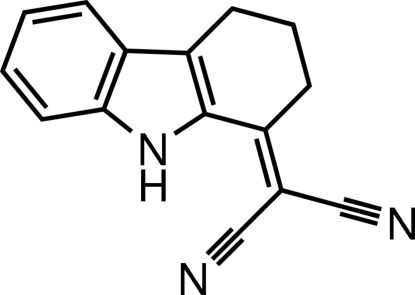


## Experimental

### 

#### Crystal data

C_15_H_11_N_3_
*M* *_r_* = 233.27
Monoclinic, 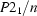

*a* = 8.4794 (3) Å
*b* = 10.5542 (4) Å
*c* = 13.0575 (5) Å
β = 97.366 (3)°
*V* = 1158.92 (8) Å^3^
*Z* = 4
Mo *K*α radiation
μ = 0.08 mm^−1^
*T* = 110 K
0.53 × 0.38 × 0.31 mm

#### Data collection

Oxford Diffraction Xcalibur Ruby Gemini diffractometer
Absorption correction: multi-scan (*CrysAlis PRO*; Oxford Diffraction, 2009 ▶) *T*_min_ = 0.939, *T*_max_ = 1.000
8311 measured reflections
3822 independent reflections
2854 reflections with *I* > 2σ(*I*)
*R*_int_ = 0.021

#### Refinement

*R*[*F* ^2^ > 2σ(*F* ^2^)] = 0.043
*wR*(*F* ^2^) = 0.115
*S* = 0.98
3822 reflections
167 parameters
H atoms treated by a mixture of independent and constrained refinement
Δρ_max_ = 0.40 e Å^−3^
Δρ_min_ = −0.24 e Å^−3^

### 

Data collection: *CrysAlis PRO* (Oxford Diffraction, 2009 ▶); cell refinement: *CrysAlis PRO*; data reduction: *CrysAlis PRO*; program(s) used to solve structure: *SHELXS97* (Sheldrick, 2008 ▶); program(s) used to refine structure: *SHELXL97* (Sheldrick, 2008 ▶); molecular graphics: *ORTEP-3* (Farrugia, 1997 ▶); software used to prepare material for publication: *PLATON* (Spek, 2009 ▶).

## Supplementary Material

Crystal structure: contains datablocks global, I. DOI: 10.1107/S1600536810022671/dn2576sup1.cif

Structure factors: contains datablocks I. DOI: 10.1107/S1600536810022671/dn2576Isup2.hkl

Additional supplementary materials:  crystallographic information; 3D view; checkCIF report

## Figures and Tables

**Table 1:** Hydrogen-bond geometry (Å, °) *Cg*1 is the centroid of the C4*B*,C5–C8,C8*A* ring.

*D*—H⋯*A*	*D*—H	H⋯*A*	*D*⋯*A*	*D*—H⋯*A*
N9—H9⋯N13	0.913 (14)	2.508 (14)	3.2626 (12)	140.3 (11)
N9—H9⋯N13^i^	0.913 (14)	2.553 (14)	3.2267 (12)	131.1 (11)
C2—H2*A*⋯*Cg*1^ii^	0.99	2.79	3.6244 (10)	142

Symmetry codes: (i) 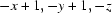; (ii) 
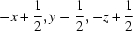
.

**Fig. 1.**
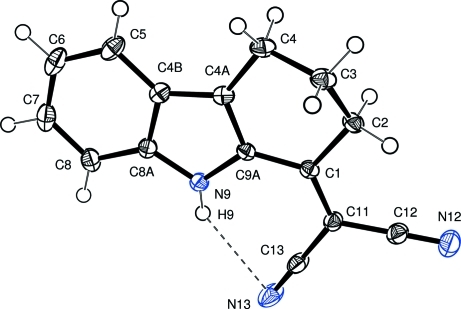
The molecular structure of the title compound, showing the atom-numbering scheme and displacement ellipsoids drawn at the 50% probability level. H atoms are shown as small spheres of arbitrary radius.

**Fig. 2.**
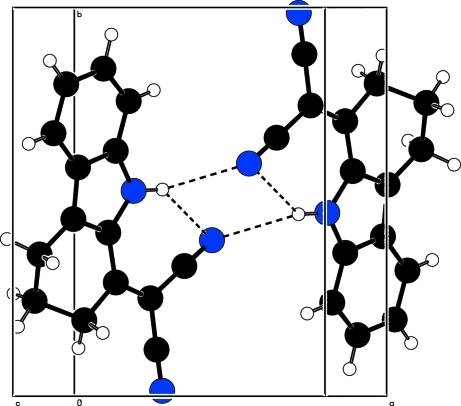
A part of the crystal structure of (I), viewed along c axis, showing the formation of a R22(14) ring.

## supplementary crystallographic information

### Comment

Tetrahydrocarbazolones have been used extensively as advanced intermediates in synthetic efforts toward a number of naturally occurring carbazole alkaloids (Scott *et al.*, 2006). Carbazole alkaloids possess various biological activities such as anti-tumor, anti-oxidative, anti-mutagenic, and anti-inflammatory activities (Ramsewak *et al.*, 1999; Tachibana *et al.*, 2001; Nakahara *et al.*, 2002). Since it is known that carbazole alkaloids possess anti-tumor activity, the identification of alkaloids that are cytotoxic against tumor cells could lead to the development of a chemopreventive agent for tumor treatment.

Gunaseelan *et al.* (2007*a*,b), Gunaseelan *et al.* (2009), Thiruvalluvar *et al.* (2007) and Sridharan *et al.* (2008) have reported the crystal structures of substituted carbazole derivatives, in which the carbazole units are not planar. In the title molecule (Scheme I, Fig. 1), C_15_H_11_N_3_, the carbazole unit is not planar. The dihedral angle between the benzene ring and the fused pyrrole ring is 1.07 (5)°. The r.m.s. deviation of a mean plane fitted through all non hydrogen atoms excluding C3 of the carbazole unit is 0.0263 Å; C3 deviates from this plane by 0.576 (1) Å. The cyclohexene ring adopts an envelope conformation. The puckering parameters (Cremer & Pople, 1975) are q2=0.3482 (10) Å, q3=-0.2564 (10) Å, Q=0.4324 (10) Å, θ=126.37 (13)° and φ=293.46 (16)°. The dicyanomethylene group at position 1 has a coplanar orientation. An intramolecular hydrogen contact N9—H9···N13 generates a ring of graph-set motif S(7) (Bernstein *et al.*, 1995)(Table 1, Fig. 1). Intermolecular N9—H9···N13 hydrogen bonds form a *R*^2^_2_(14)(Bernstein *et al.*, 1995) ring in the crystal structure (Table 1, Fig. 2). A C2—H2A···π interaction involving the benzene (C4B,C5—C8,C8A) ring is also found in the structure(Table 1).

### Experimental

A mixture of 2,3,4,9-tetrahydro-1*H*-carbazol-1-one (0.199 g, 0.001 mol), malononitrile (0.066 g, 0.001 mol), ammonium acetate (0.092 g, 0.0012 mol) and few drops of acetic acid in 5 ml of toluene was refluxed at 383 K for 6 h. On cooling, the precipitate that formed was filtered off, washed with petroleum ether and dried. The crude product thus obtained was purified by column chromatography over silica gel using petroleum ether: ethyl acetate (99:1, *v*/*v*) to yield the titled product (0.173 g, 74%). This was recrystallized from ethyl acetate.

### Refinement

The H atom bonded to N9 was located in a difference Fourier map and refined freely. Other H atoms were positioned geometrically and allowed to ride on their parent atoms, with C—H = 0.95–0.99 Å and *U*_iso_(H) = 1.2*U*_eq_(parent atom).

### Crystal data

**Table:**

C_15_H_11_N_3_	*F*(000) = 488
*M**_r_* = 233.27	*D*_x_ = 1.337 Mg m^−^^3^
Monoclinic, *P*2_1_/*n*	Melting point: 470 K
Hall symbol: -P 2yn	Mo *K*α radiation, λ = 0.71073 Å
*a* = 8.4794 (3) Å	Cell parameters from 4130 reflections
*b* = 10.5542 (4) Å	θ = 4.7–32.6°
*c* = 13.0575 (5) Å	µ = 0.08 mm^−^^1^
β = 97.366 (3)°	*T* = 110 K
*V* = 1158.92 (8) Å^3^	Prism, pale-yellow
*Z* = 4	0.53 × 0.38 × 0.31 mm

### Data collection

**Table:**

Oxford Diffraction Xcalibur Ruby Gemini diffractometer	3822 independent reflections
Radiation source: Enhance (Mo) X-ray Source	2854 reflections with *I* > 2σ(*I*)
graphite	*R*_int_ = 0.021
Detector resolution: 10.5081 pixels mm^-1^	θ_max_ = 32.6°, θ_min_ = 4.7°
ω scans	*h* = −12→12
Absorption correction: multi-scan (*CrysAlis PRO*; Oxford Diffraction, 2009)	*k* = −15→15
*T*_min_ = 0.939, *T*_max_ = 1.000	*l* = −19→16
8311 measured reflections	

### Refinement

**Table:**

Refinement on *F*^2^	Primary atom site location: structure-invariant direct methods
Least-squares matrix: full	Secondary atom site location: difference Fourier map
*R*[*F*^2^ > 2σ(*F*^2^)] = 0.043	Hydrogen site location: inferred from neighbouring sites
*wR*(*F*^2^) = 0.115	H atoms treated by a mixture of independent and constrained refinement
*S* = 0.98	*w* = 1/[σ^2^(*F*_o_^2^) + (0.0736*P*)^2^] where *P* = (*F*_o_^2^ + 2*F*_c_^2^)/3
3822 reflections	(Δ/σ)_max_ = 0.001
167 parameters	Δρ_max_ = 0.40 e Å^−^^3^
0 restraints	Δρ_min_ = −0.24 e Å^−^^3^

### Special details

**Table:**

Geometry. Bond distances, angles *etc*. have been calculated using the rounded fractional coordinates. All su's are estimated from the variances of the (full) variance-covariance matrix. The cell e.s.d.'s are taken into account in the estimation of distances, angles and torsion angles
Refinement. Refinement of *F*^2^ against ALL reflections. The weighted *R*-factor *wR* and goodness of fit *S* are based on *F*^2^, conventional *R*-factors *R* are based on *F*, with *F* set to zero for negative *F*^2^. The threshold expression of *F*^2^ > 2σ(*F*^2^) is used only for calculating *R*-factors(gt) *etc*. and is not relevant to the choice of reflections for refinement. *R*-factors based on *F*^2^ are statistically about twice as large as those based on *F*, and *R*- factors based on ALL data will be even larger.

### Fractional atomic coordinates and isotropic or equivalent isotropic displacement parameters (Å^2^)

**Table:**

	*x*	*y*	*z*	*U*_iso_*/*U*_eq_	
N9	0.20402 (9)	0.52842 (7)	0.07142 (6)	0.0170 (2)	
N12	0.27359 (12)	0.01410 (9)	−0.03875 (8)	0.0333 (3)	
N13	0.42310 (11)	0.40137 (9)	−0.08917 (7)	0.0351 (3)	
C1	0.14687 (10)	0.29269 (9)	0.07178 (6)	0.0152 (2)	
C2	0.05668 (11)	0.19571 (9)	0.12634 (7)	0.0194 (2)	
C3	−0.09527 (10)	0.24541 (10)	0.16360 (7)	0.0219 (3)	
C4	−0.06619 (11)	0.36476 (10)	0.22890 (7)	0.0212 (3)	
C4A	0.03186 (10)	0.45589 (9)	0.17735 (6)	0.0166 (2)	
C4B	0.04943 (10)	0.58875 (9)	0.19177 (7)	0.0179 (2)	
C5	−0.01638 (11)	0.67652 (10)	0.25578 (8)	0.0238 (3)	
C6	0.02314 (12)	0.80254 (10)	0.24880 (8)	0.0275 (3)	
C7	0.12868 (12)	0.84267 (10)	0.18004 (8)	0.0264 (3)	
C8	0.19716 (11)	0.75911 (9)	0.11745 (7)	0.0221 (2)	
C8A	0.15696 (10)	0.63122 (9)	0.12427 (7)	0.0173 (2)	
C9A	0.12894 (10)	0.42084 (8)	0.10364 (6)	0.0151 (2)	
C11	0.24383 (10)	0.25207 (9)	0.00157 (7)	0.0178 (2)	
C12	0.25910 (11)	0.12004 (10)	−0.02084 (7)	0.0222 (3)	
C13	0.34255 (11)	0.33496 (10)	−0.04916 (7)	0.0226 (2)	
H2A	0.12768	0.16285	0.18658	0.0232*	
H2B	0.02872	0.12377	0.07880	0.0232*	
H3A	−0.17457	0.26402	0.10301	0.0262*	
H3B	−0.14007	0.17888	0.20485	0.0262*	
H4A	−0.16918	0.40435	0.23835	0.0255*	
H4B	−0.01056	0.34246	0.29787	0.0255*	
H5	−0.08640	0.64936	0.30266	0.0286*	
H6	−0.02115	0.86313	0.29074	0.0330*	
H7	0.15343	0.93025	0.17673	0.0317*	
H8	0.26848	0.78727	0.07176	0.0265*	
H9	0.2890 (16)	0.5317 (13)	0.0351 (11)	0.043 (4)*	

### Atomic displacement parameters (Å^2^)

**Table:**

	*U*^11^	*U*^22^	*U*^33^	*U*^12^	*U*^13^	*U*^23^
N9	0.0188 (3)	0.0158 (4)	0.0172 (3)	−0.0019 (3)	0.0052 (3)	−0.0004 (3)
N12	0.0414 (5)	0.0225 (4)	0.0389 (5)	−0.0017 (4)	0.0158 (4)	−0.0055 (4)
N13	0.0396 (5)	0.0323 (5)	0.0383 (5)	−0.0131 (4)	0.0240 (4)	−0.0134 (4)
C1	0.0148 (4)	0.0166 (4)	0.0140 (4)	−0.0015 (3)	0.0007 (3)	0.0009 (3)
C2	0.0228 (4)	0.0177 (4)	0.0181 (4)	−0.0047 (3)	0.0046 (3)	0.0012 (4)
C3	0.0198 (4)	0.0262 (5)	0.0205 (4)	−0.0062 (4)	0.0057 (3)	0.0006 (4)
C4	0.0185 (4)	0.0271 (5)	0.0194 (4)	−0.0021 (4)	0.0074 (3)	−0.0002 (4)
C4A	0.0144 (4)	0.0204 (4)	0.0149 (4)	0.0006 (3)	0.0018 (3)	0.0004 (3)
C4B	0.0149 (4)	0.0208 (4)	0.0176 (4)	0.0022 (3)	0.0002 (3)	−0.0015 (4)
C5	0.0179 (4)	0.0290 (5)	0.0240 (4)	0.0059 (4)	0.0007 (3)	−0.0076 (4)
C6	0.0250 (5)	0.0263 (5)	0.0293 (5)	0.0096 (4)	−0.0040 (4)	−0.0107 (4)
C7	0.0292 (5)	0.0179 (4)	0.0290 (5)	0.0037 (4)	−0.0081 (4)	−0.0037 (4)
C8	0.0255 (4)	0.0175 (4)	0.0218 (4)	−0.0003 (4)	−0.0028 (3)	0.0007 (4)
C8A	0.0181 (4)	0.0169 (4)	0.0160 (4)	0.0014 (3)	−0.0013 (3)	−0.0009 (3)
C9A	0.0153 (4)	0.0157 (4)	0.0143 (4)	−0.0015 (3)	0.0023 (3)	0.0013 (3)
C11	0.0189 (4)	0.0166 (4)	0.0183 (4)	−0.0025 (3)	0.0044 (3)	−0.0026 (3)
C12	0.0238 (4)	0.0220 (5)	0.0219 (4)	−0.0014 (4)	0.0067 (3)	−0.0026 (4)
C13	0.0236 (4)	0.0221 (4)	0.0238 (4)	−0.0037 (4)	0.0097 (4)	−0.0081 (4)

### Geometric parameters (Å, °)

**Table:**

N9—C8A	1.3723 (12)	C6—C7	1.4115 (15)
N9—C9A	1.3929 (11)	C7—C8	1.3801 (14)
N12—C12	1.1521 (14)	C8—C8A	1.3978 (13)
N13—C13	1.1499 (14)	C11—C12	1.4331 (14)
N9—H9	0.913 (14)	C11—C13	1.4307 (13)
C1—C2	1.5099 (13)	C2—H2A	0.9900
C1—C11	1.3760 (12)	C2—H2B	0.9900
C1—C9A	1.4289 (13)	C3—H3A	0.9900
C2—C3	1.5273 (13)	C3—H3B	0.9900
C3—C4	1.5234 (14)	C4—H4A	0.9900
C4—C4A	1.4876 (13)	C4—H4B	0.9900
C4A—C9A	1.3943 (12)	C5—H5	0.9500
C4A—C4B	1.4201 (13)	C6—H6	0.9500
C4B—C5	1.4099 (14)	C7—H7	0.9500
C4B—C8A	1.4196 (13)	C8—H8	0.9500
C5—C6	1.3775 (15)		
			
N9···N13	3.2626 (12)	C8A···H2A^vii^	2.9000
N9···C13	2.9158 (13)	C9A···H3A	3.0600
N9···N13^i^	3.2267 (12)	C11···H9	3.001 (14)
N9···C9A^ii^	3.4392 (11)	C12···H2A	3.0900
N12···C3^iii^	3.4371 (14)	C12···H2B	2.4800
N13···N9	3.2626 (12)	C13···H9	2.420 (14)
N13···N9^i^	3.2267 (12)	H2A···C12	3.0900
N13···N13^i^	3.2679 (13)	H2A···H7^iv^	2.4700
N12···H2B^iii^	2.9400	H2A···C4B^vi^	3.0800
N12···H8^iv^	2.8000	H2A···C8^vi^	2.9700
N13···H9	2.508 (14)	H2A···C8A^vi^	2.9000
N13···H9^i^	2.553 (14)	H2B···C12	2.4800
N13···H3B^v^	2.8100	H2B···H7^iv^	2.5600
C1···C6^vi^	3.4129 (13)	H2B···N12^iii^	2.9400
C1···C7^vi^	3.5806 (13)	H3A···C9A	3.0600
C1···C8A^ii^	3.4849 (12)	H3A···C8^ii^	2.8700
C3···N12^iii^	3.4371 (14)	H3A···H8^ii^	2.3800
C4A···C7^vi^	3.4360 (13)	H3B···C5^viii^	3.0200
C6···C9A^vii^	3.5380 (13)	H3B···H5^viii^	2.3300
C6···C1^vii^	3.4129 (13)	H3B···N13^x^	2.8100
C7···C9A^vii^	3.3756 (13)	H4B···C8^vi^	2.8800
C7···C1^vii^	3.5806 (13)	H4B···H8^vi^	2.5600
C7···C4A^vii^	3.4360 (13)	H5···C3^ix^	2.9700
C8A···C1^ii^	3.4849 (12)	H5···H3B^ix^	2.3300
C9A···N9^ii^	3.4392 (11)	H7···C2^xi^	2.9700
C9A···C6^vi^	3.5380 (13)	H7···H2A^xi^	2.4700
C9A···C7^vi^	3.3756 (13)	H7···H2B^xi^	2.5600
C13···N9	2.9158 (13)	H7···C4A^vii^	3.0900
C2···H7^iv^	2.9700	H8···N12^xi^	2.8000
C3···H5^viii^	2.9700	H8···H4B^vii^	2.5600
C4A···H7^vi^	3.0900	H8···H3A^ii^	2.3800
C4B···H2A^vii^	3.0800	H9···N13	2.508 (14)
C5···H3B^ix^	3.0200	H9···C11	3.001 (14)
C8···H3A^ii^	2.8700	H9···C13	2.420 (14)
C8···H2A^vii^	2.9700	H9···N13^i^	2.553 (14)
C8···H4B^vii^	2.8800		
			
C8A—N9—C9A	108.60 (7)	C12—C11—C13	115.24 (8)
C9A—N9—H9	127.6 (9)	N12—C12—C11	179.07 (10)
C8A—N9—H9	122.1 (9)	N13—C13—C11	179.33 (10)
C2—C1—C11	119.03 (8)	C1—C2—H2A	109.00
C2—C1—C9A	115.16 (7)	C1—C2—H2B	109.00
C9A—C1—C11	125.72 (8)	C3—C2—H2A	109.00
C1—C2—C3	114.61 (8)	C3—C2—H2B	109.00
C2—C3—C4	112.31 (8)	H2A—C2—H2B	108.00
C3—C4—C4A	109.95 (7)	C2—C3—H3A	109.00
C4—C4A—C9A	123.68 (8)	C2—C3—H3B	109.00
C4B—C4A—C9A	107.00 (8)	C4—C3—H3A	109.00
C4—C4A—C4B	129.32 (8)	C4—C3—H3B	109.00
C5—C4B—C8A	119.68 (9)	H3A—C3—H3B	108.00
C4A—C4B—C5	133.21 (8)	C3—C4—H4A	110.00
C4A—C4B—C8A	107.11 (8)	C3—C4—H4B	110.00
C4B—C5—C6	118.49 (9)	C4A—C4—H4A	110.00
C5—C6—C7	120.75 (10)	C4A—C4—H4B	110.00
C6—C7—C8	122.29 (10)	H4A—C4—H4B	108.00
C7—C8—C8A	117.05 (9)	C4B—C5—H5	121.00
N9—C8A—C4B	108.30 (8)	C6—C5—H5	121.00
N9—C8A—C8	129.97 (8)	C5—C6—H6	120.00
C4B—C8A—C8	121.72 (8)	C7—C6—H6	120.00
N9—C9A—C1	127.87 (8)	C6—C7—H7	119.00
C1—C9A—C4A	123.12 (8)	C8—C7—H7	119.00
N9—C9A—C4A	108.99 (8)	C7—C8—H8	121.00
C1—C11—C13	123.57 (9)	C8A—C8—H8	121.00
C1—C11—C12	121.09 (8)		
			
C9A—N9—C8A—C4B	−0.03 (12)	C4—C4A—C4B—C8A	179.76 (8)
C9A—N9—C8A—C8	−178.67 (9)	C9A—C4A—C4B—C5	−179.41 (10)
C8A—N9—C9A—C1	−177.78 (8)	C9A—C4A—C4B—C8A	0.72 (10)
C8A—N9—C9A—C4A	0.48 (10)	C4—C4A—C9A—N9	−179.85 (8)
C9A—C1—C2—C3	28.90 (11)	C4—C4A—C9A—C1	−1.49 (13)
C11—C1—C2—C3	−154.52 (8)	C4B—C4A—C9A—N9	−0.75 (9)
C2—C1—C9A—N9	176.14 (8)	C4B—C4A—C9A—C1	177.62 (8)
C2—C1—C9A—C4A	−1.90 (12)	C4A—C4B—C5—C6	−178.23 (10)
C11—C1—C9A—N9	−0.17 (14)	C8A—C4B—C5—C6	1.62 (14)
C11—C1—C9A—C4A	−178.21 (8)	C4A—C4B—C8A—N9	−0.44 (10)
C2—C1—C11—C12	0.52 (13)	C4A—C4B—C8A—C8	178.34 (8)
C2—C1—C11—C13	−175.65 (8)	C5—C4B—C8A—N9	179.67 (8)
C9A—C1—C11—C12	176.71 (8)	C5—C4B—C8A—C8	−1.55 (14)
C9A—C1—C11—C13	0.54 (14)	C4B—C5—C6—C7	−0.78 (15)
C1—C2—C3—C4	−52.67 (10)	C5—C6—C7—C8	−0.22 (16)
C2—C3—C4—C4A	46.84 (10)	C6—C7—C8—C8A	0.34 (15)
C3—C4—C4A—C4B	159.52 (9)	C7—C8—C8A—N9	179.04 (9)
C3—C4—C4A—C9A	−21.59 (12)	C7—C8—C8A—C4B	0.55 (14)
C4—C4A—C4B—C5	−0.37 (17)		

Symmetry codes: (i) −*x*+1, −*y*+1, −*z*; (ii) −*x*, −*y*+1, −*z*; (iii) −*x*, −*y*, −*z*; (iv) *x*, *y*−1, *z*; (v) *x*+1/2, −*y*+1/2, *z*−1/2; (vi) −*x*+1/2, *y*−1/2, −*z*+1/2; (vii) −*x*+1/2, *y*+1/2, −*z*+1/2; (viii) −*x*−1/2, *y*−1/2, −*z*+1/2; (ix) −*x*−1/2, *y*+1/2, −*z*+1/2; (x) *x*−1/2, −*y*+1/2, *z*+1/2; (xi) *x*, *y*+1, *z*.

### Hydrogen-bond geometry (Å, °)

**Table:**

Cg1 is the centroid of the C4B,C5–C8,C8A ring.

**Table:**

*D*—H···*A*	*D*—H	H···*A*	*D*···*A*	*D*—H···*A*
N9—H9···N13	0.913 (14)	2.508 (14)	3.2626 (12)	140.3 (11)
N9—H9···N13^i^	0.913 (14)	2.553 (14)	3.2267 (12)	131.1 (11)
C2—H2A···Cg1^vi^	0.99	2.79	3.6244 (10)	142

Symmetry codes: (i) −*x*+1, −*y*+1, −*z*; (vi) −*x*+1/2, *y*−1/2, −*z*+1/2.

## References

[bb1] Bernstein, J., Davis, R. E., Shimoni, L. & Chang, N.-L. (1995). *Angew. Chem. Int. Ed. Engl.* **34**, 1555–1573.

[bb2] Cremer, D. & Pople, J. A. (1975). *J. Am. Chem. Soc.* **97**, 1354–1358.

[bb3] Farrugia, L. J. (1997). *J. Appl. Cryst.* **30**, 565.

[bb4] Gunaseelan, A. T., Prabakaran, K., Prasad, K. J. R., Thiruvalluvar, A. & Butcher, R. J. (2009). *Acta Cryst.* E**65**, o1946–o1947. 10.1107/S1600536809028050 PMC2977477 21583627

[bb5] Gunaseelan, A. T., Thiruvalluvar, A., Martin, A. E. & Prasad, K. J. R. (2007*a*). *Acta Cryst.* E**63**, o2413–o2414.

[bb6] Gunaseelan, A. T., Thiruvalluvar, A., Martin, A. E. & Prasad, K. J. R. (2007*b*). *Acta Cryst.* E**63**, o2729–o2730.

[bb7] Nakahara, K., Trakoontivakorn, G., Alzoreky, N. S., Ono, H., Onishi-Kameyama, M. & Yoshida, M. (2002). *J. Agric. Food Chem.* **50**, 4796–4802. 10.1021/jf025564w 12166962

[bb8] Oxford Diffraction (2009). *CrysAlis PRO* Oxford Diffraction Ltd, Yarnton, England.

[bb9] Ramsewak, R. S., Nair, M. G., Strasburg, G. M., DeWitt, D. L. & Nitiss, J. L. (1999). *J. Agric. Food Chem.* **47**, 444–447. 10.1021/jf9805808 10563914

[bb10] Scott, T. L., Yu, X., Gorugantula, S. P., Carrero-Martinez, G. & Söderberg, B. C. G. (2006). *Tetrahedron*, **62**, 10835–10842.

[bb11] Sheldrick, G. M. (2008). *Acta Cryst.* A**64**, 112–122. 10.1107/S0108767307043930 18156677

[bb12] Spek, A. L. (2009). *Acta Cryst.* D**65**, 148–155. 10.1107/S090744490804362X PMC2631630 19171970

[bb13] Sridharan, M., Prasad, K. J. R., Gunaseelan, A. T., Thiruvalluvar, A. & Linden, A. (2008). *Acta Cryst.* E**64**, o763–o764. 10.1107/S1600536808007885 PMC2960955 21202152

[bb14] Tachibana, Y., Kikuzaki, H., Lajis, N. H. & Nakatani, N. (2001). *J. Agric. Food Chem.* **49**, 5589–5594. 10.1021/jf010621r 11714364

[bb15] Thiruvalluvar, A., Gunaseelan, A. T., Martin, A. E., Prasad, K. J. R. & Butcher, R. J. (2007). *Acta Cryst.* E**63**, o3524.

